# Supplementary figures and images for: Predicting bacterial growth conditions from mRNA and protein abundances
Source: PLoS One. 2018 Nov 2;13(11):e0206634. doi: 10.1371/journal.pone.0206634 (PMC6214550; doi:10.1371/journal.pone.0206634)

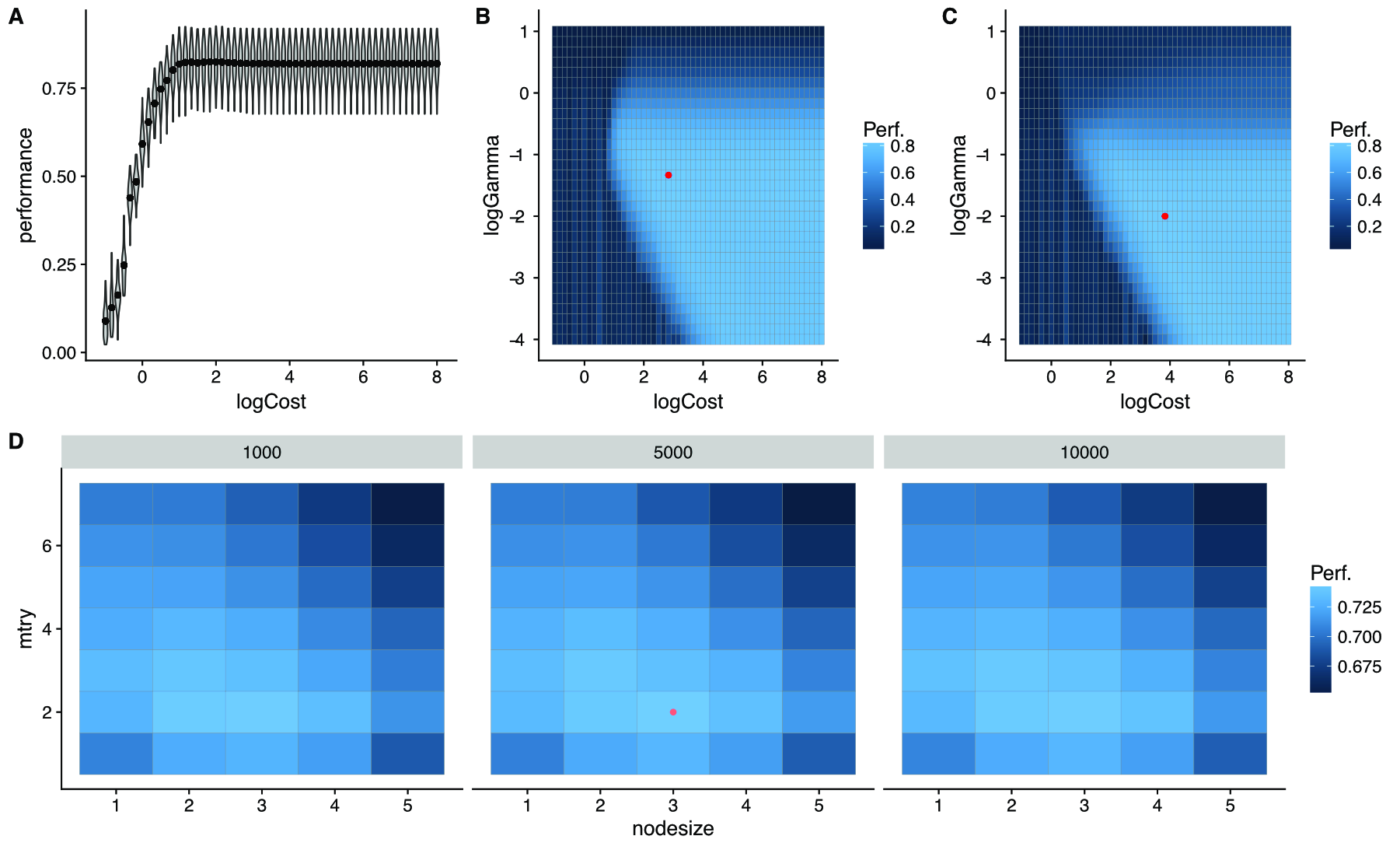

Supplement: S1 Fig — Model performance is measured as the mean F1 score over 10 independent tuning runs. Higher numbers indicate better performance. (A) Tuning results for SVMs with linear kernel. Only the cost parameter was tuned. (B) Tuning results for SVMs with radial kernel. The cost and gamma parameters were tuned. The red dot indicates the winning parameter combination. (C) Tuning results for SVMs with sigmoidal kernel. The cost and gamma parameters were tuned. The red dot indicates the winning parameter combination. (D) Tuning results for random forest models. The mtry, nodesize, and ntrees parameters were tuned. We used three values for ntrees, 1000, 5000, and 10000, shown as three separate panels. The red dot indicates the winning parameter combination. (TIFF) [file pone.0206634.s002.tiff]

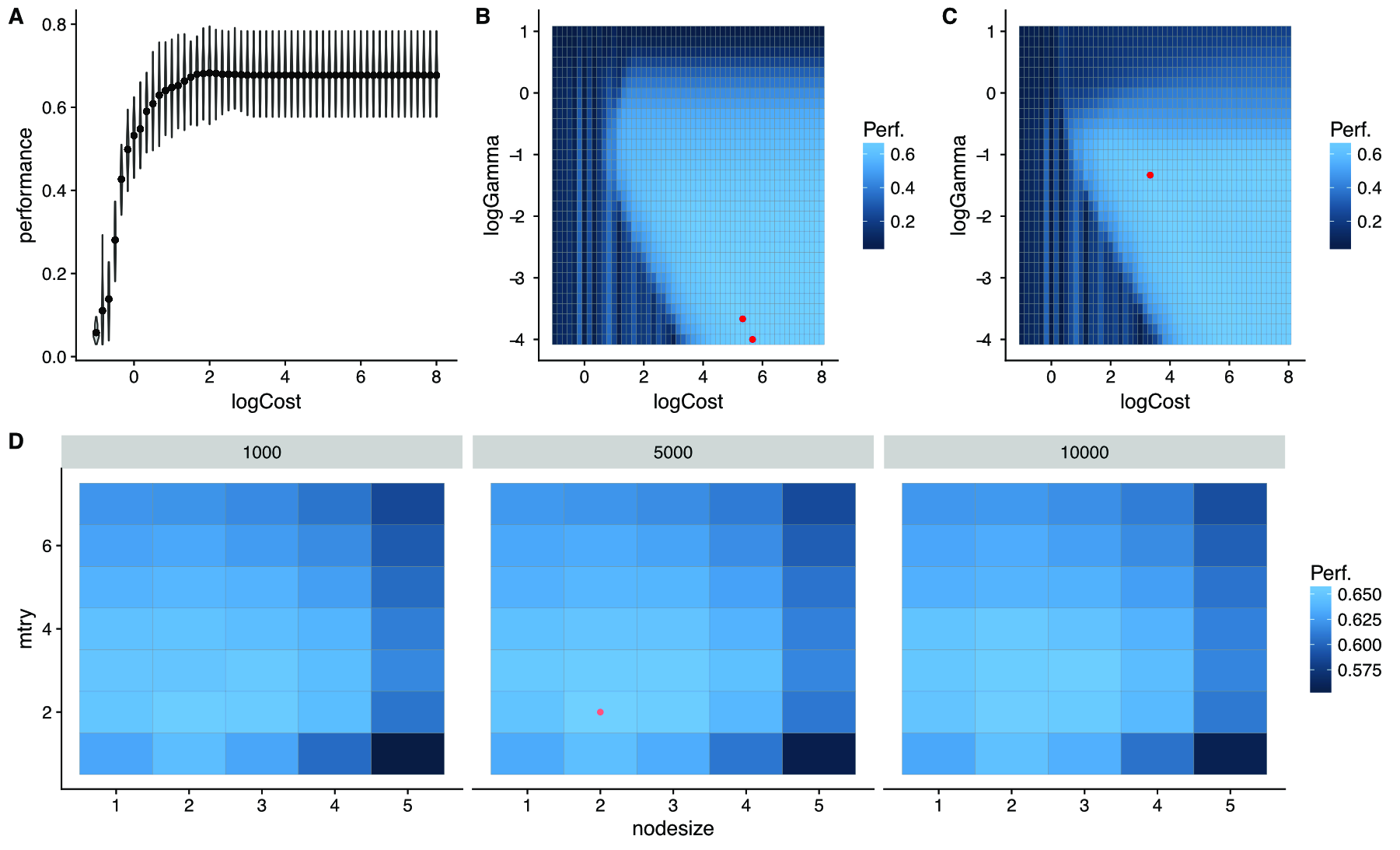

Supplement: S2 Fig — (A) Tuning results for SVMs with linear kernel. Only the cost parameter was tuned. (B) Tuning results for SVMs with radial kernel. The cost and gamma parameters were tuned. The red dots indicate the winning parameter combinations. (C) Tuning results for SVMs with sigmoidal kernel. The cost and gamma parameters were tuned. The red dot indicates the winning parameter combination. (D) Tuning results for random forest models. The mtry, nodesize, and ntrees parameters were tuned. We used three values for ntrees, 1000, 5000, and 10000, shown as three separate panels. The red dot indicates the winning parameter combination. (TIFF) [file pone.0206634.s003.tiff]

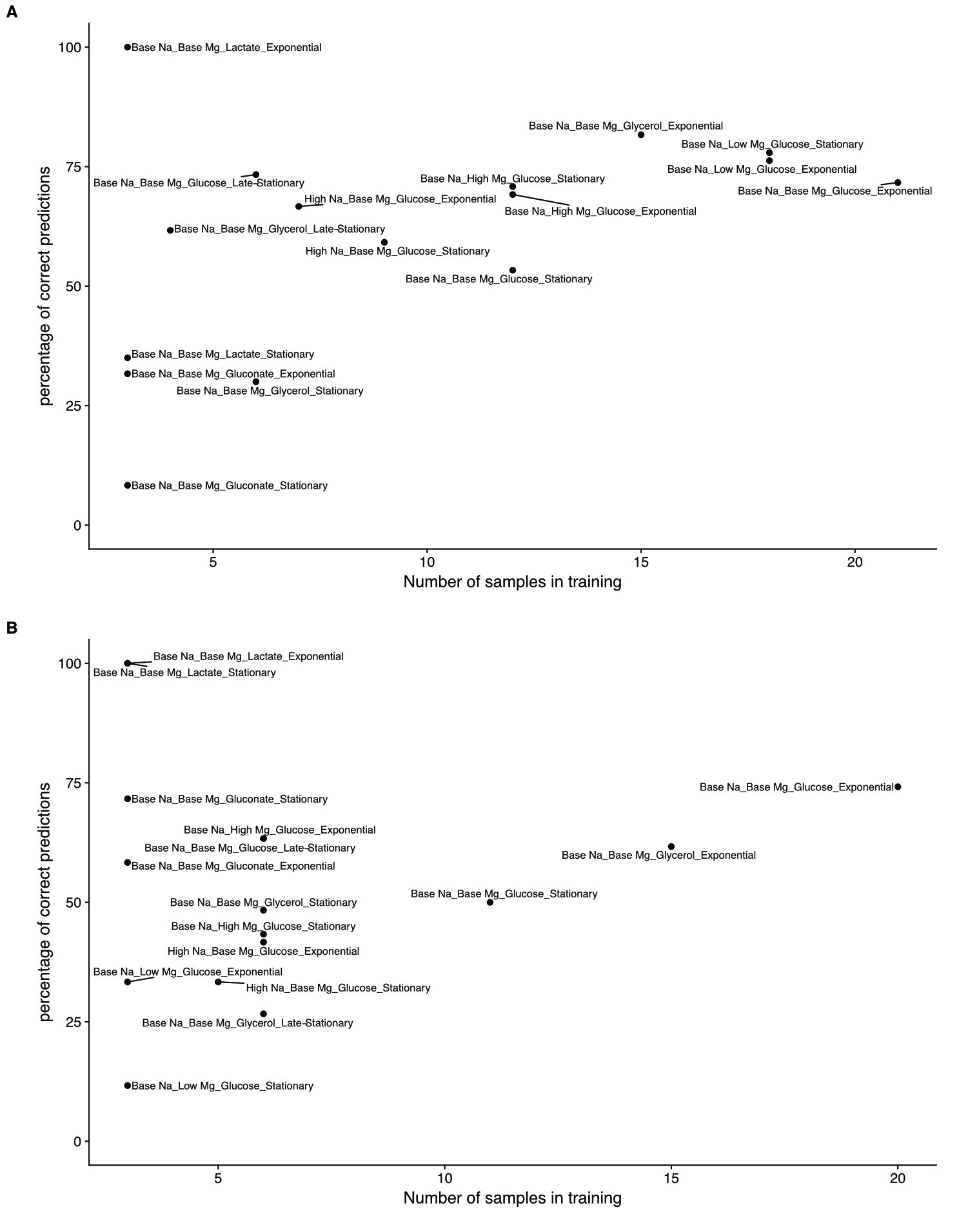

Supplement: S3 Fig — (A) Predictions based on mRNA abundances. (B) Predictions based on protein abundances. (TIFF) [file pone.0206634.s004.tiff]

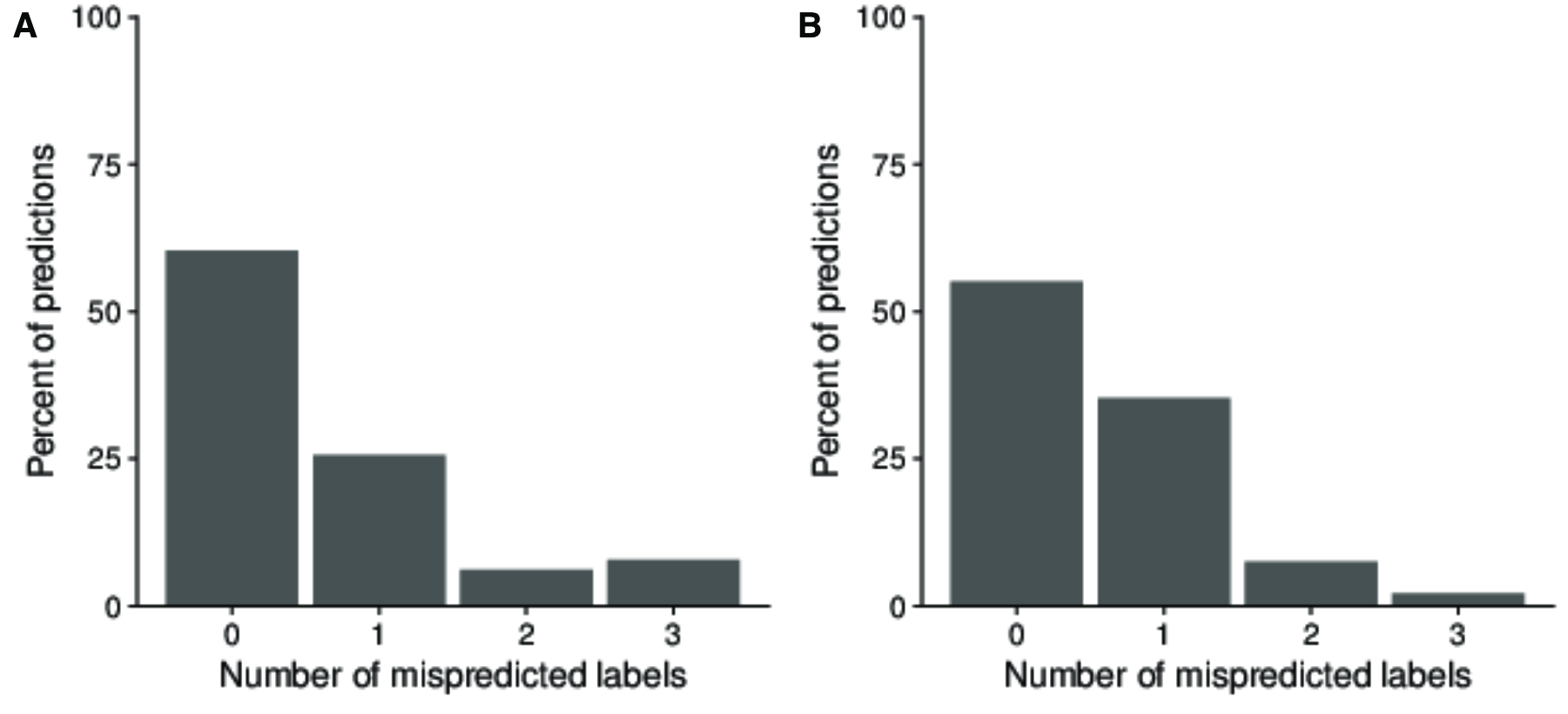

Supplement: S4 Fig — The error count distribution for mRNA (A) and protein (B) confusion matrices. The number of mis-predicted labels (x-axis) indicates how many of the 4 possible condition variables that an individual prediction got wrong. 0 mis-predicted labels (the majority in both cases) means that model predictions were 100% accurate. In both cases (mRNA and protein), when an incorrect prediction was made, it was most frequently due to a single variable being incorrectly predicted (number of mis-predicted labels with a value of 1) as compared to errors predicting more than one variable for a given condition (2 and 3 mis-predicted labels). (TIFF) [file pone.0206634.s005.tiff]

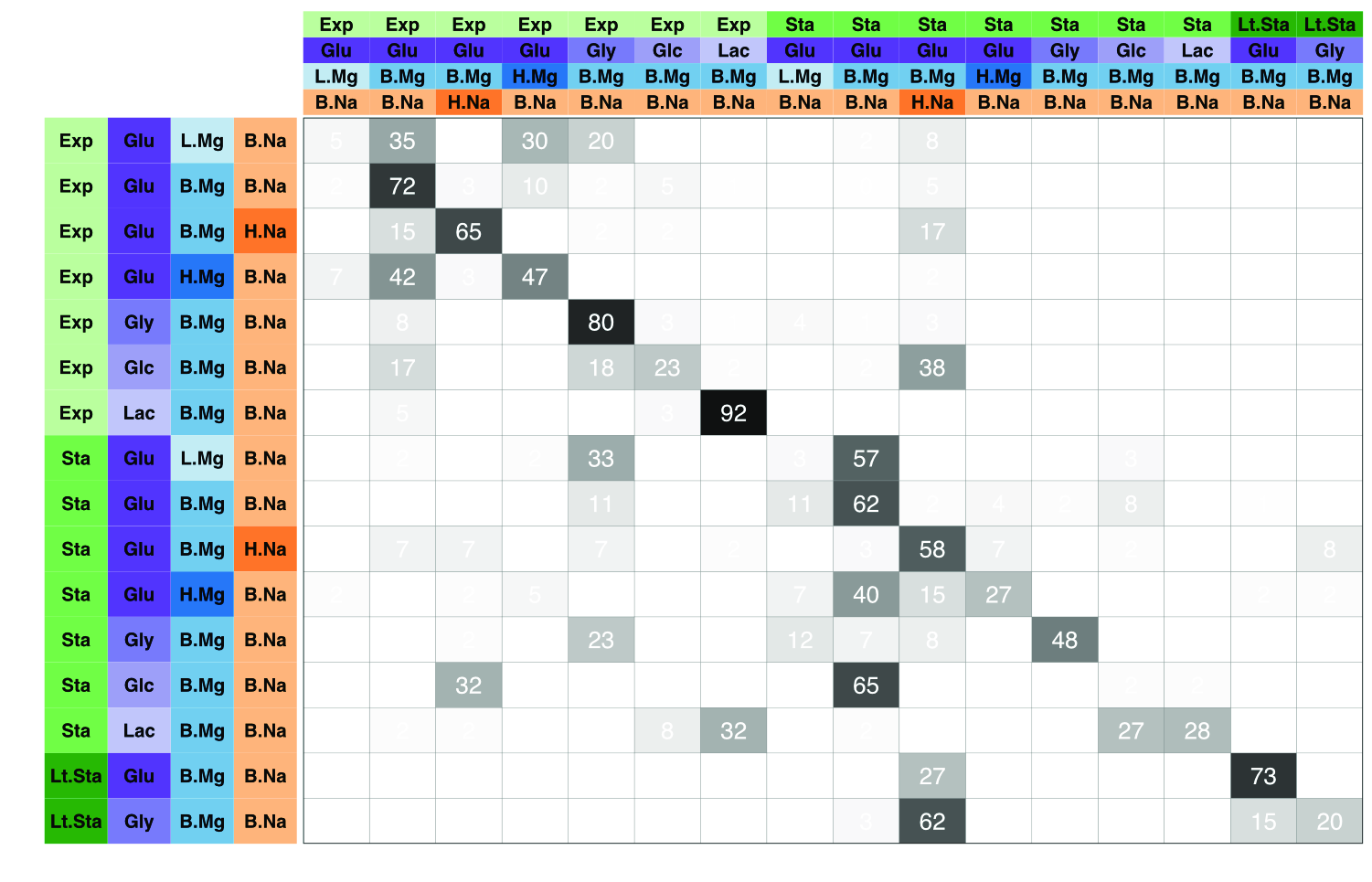

Supplement: S5 Fig — Rows represent true conditions and columns represent predicted conditions. The numbers in the cells and the shading of the cells represent the percentage (out of 60 independent replicates) with which a given true condition is predicted as a certain predicted condition. Predictions based on mRNA abundances, generated by using subset of mRNA samples which has matching protein pairs. Results are shown for the SVM with radial kernel, which was the best performing model in the tuning process on mRNA data, where it won 48 of 60 independent runs. The average of the diagonal line is 44.1% and multi class macro F1 score is 0.43. (TIFF) [file pone.0206634.s006.tiff]

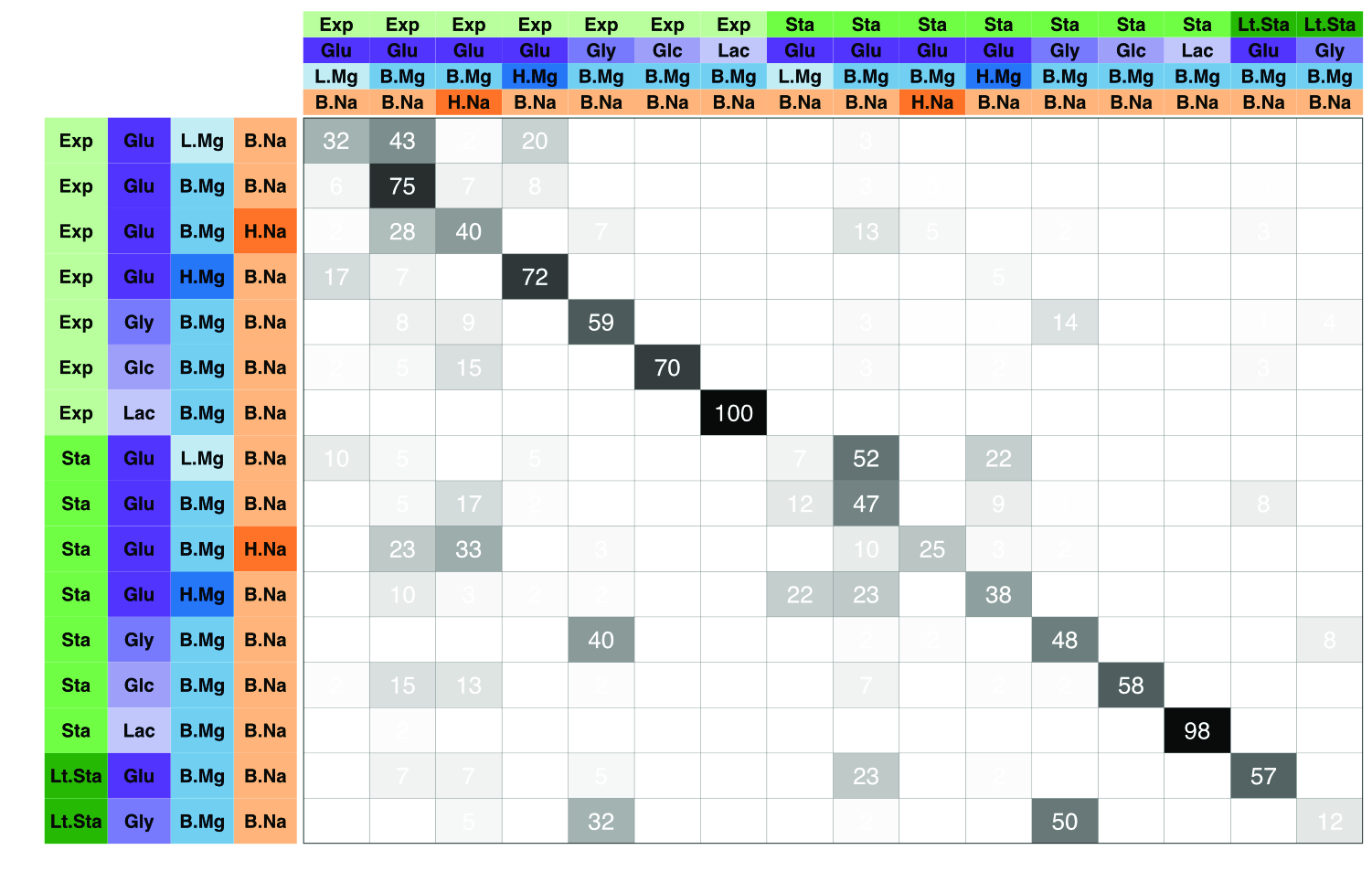

Supplement: S6 Fig — Rows represent true conditions and columns represent predicted conditions. The numbers in the cells and the shading of the cells represent the percentage (out of 60 independent replicates) with which a given true condition is predicted as a certain predicted condition. Predictions based on protein abundances, generated by using subset of protein samples which has matching mRNA pairs. Results are shown for the SVM with sigmoid kernel, which was the best performing model in the tuning process on mRNA data, where it won 47 of 60 independent runs. The average of the diagonal line is 52.3% and corresponding multi class macro F1 score is 0.53. (TIFF) [file pone.0206634.s007.tiff]

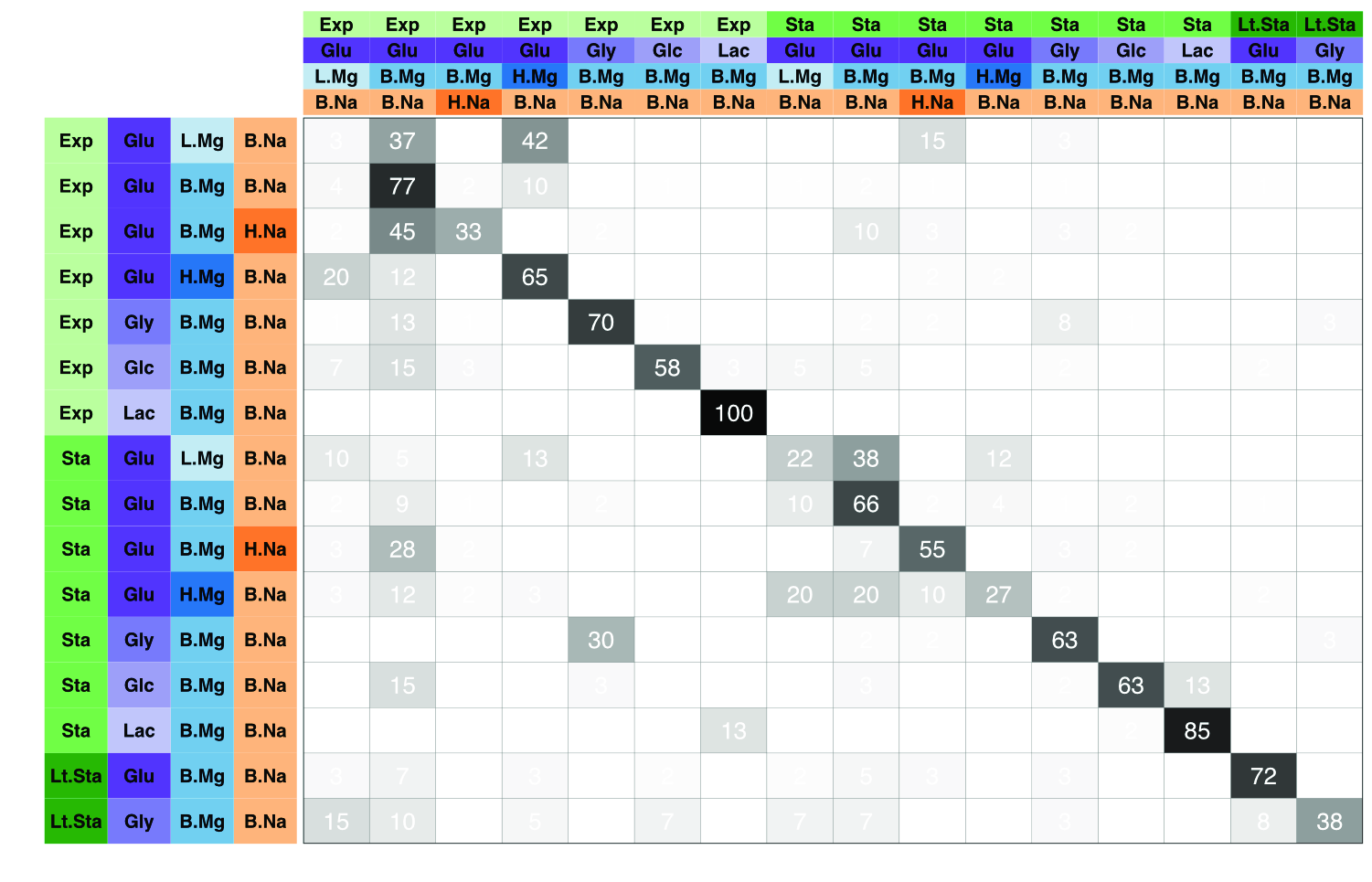

Supplement: S7 Fig — Rows represent true conditions and columns represent predicted conditions. The numbers in the cells and the shading of the cells represent the percentage (out of 60 independent replicates) with which a given true condition is predicted as a certain predicted condition. Predictions based on protein abundances, generated by using subset of mRNA & protein samples which has matching pairs. Results are shown for the SVM with sigmoid kernel, which was the best performing model in the tuning process on combined intersection data, where it won 27 of 60 independent runs. The average of the diagonal line is 56.1% and corresponding multi class macro F1 score is 0.57. (TIFF) [file pone.0206634.s008.tiff]

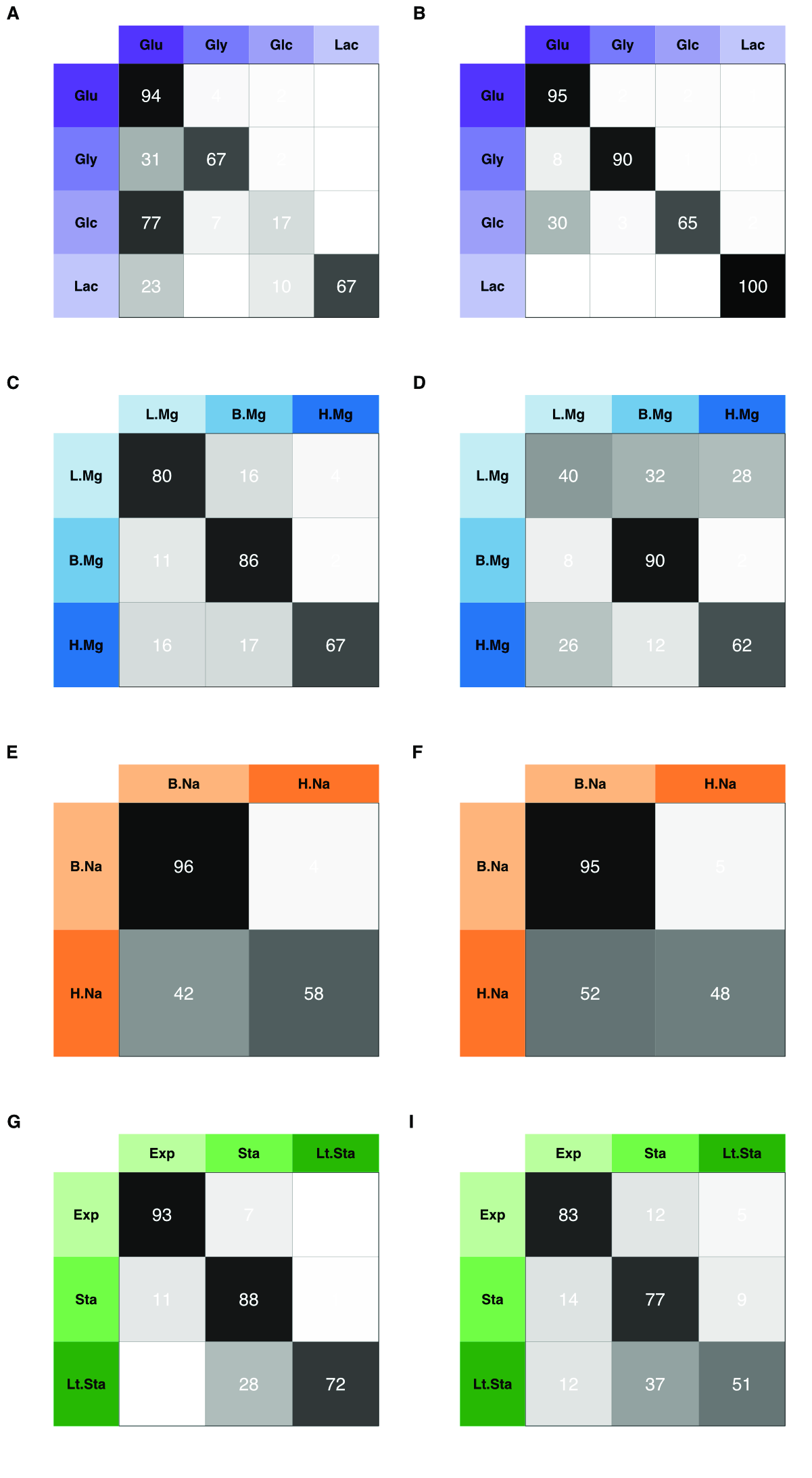

Supplement: S8 Fig — (A) Prediction of carbon source from mRNA abundances. (B) Prediction of carbon source from protein abundances. (C) Prediction of growth phase from mRNA abundances. (D) Prediction of growth phase from protein abundances. (E) Prediction of Mg2+ levels from mRNA abundances. (F) Prediction of Mg2+ levels from protein abundances. (G) Prediction of Na+ levels from mRNA abundances. (H) Prediction of Na+ levels from protein abundances. (TIFF) [file pone.0206634.s009.tiff]

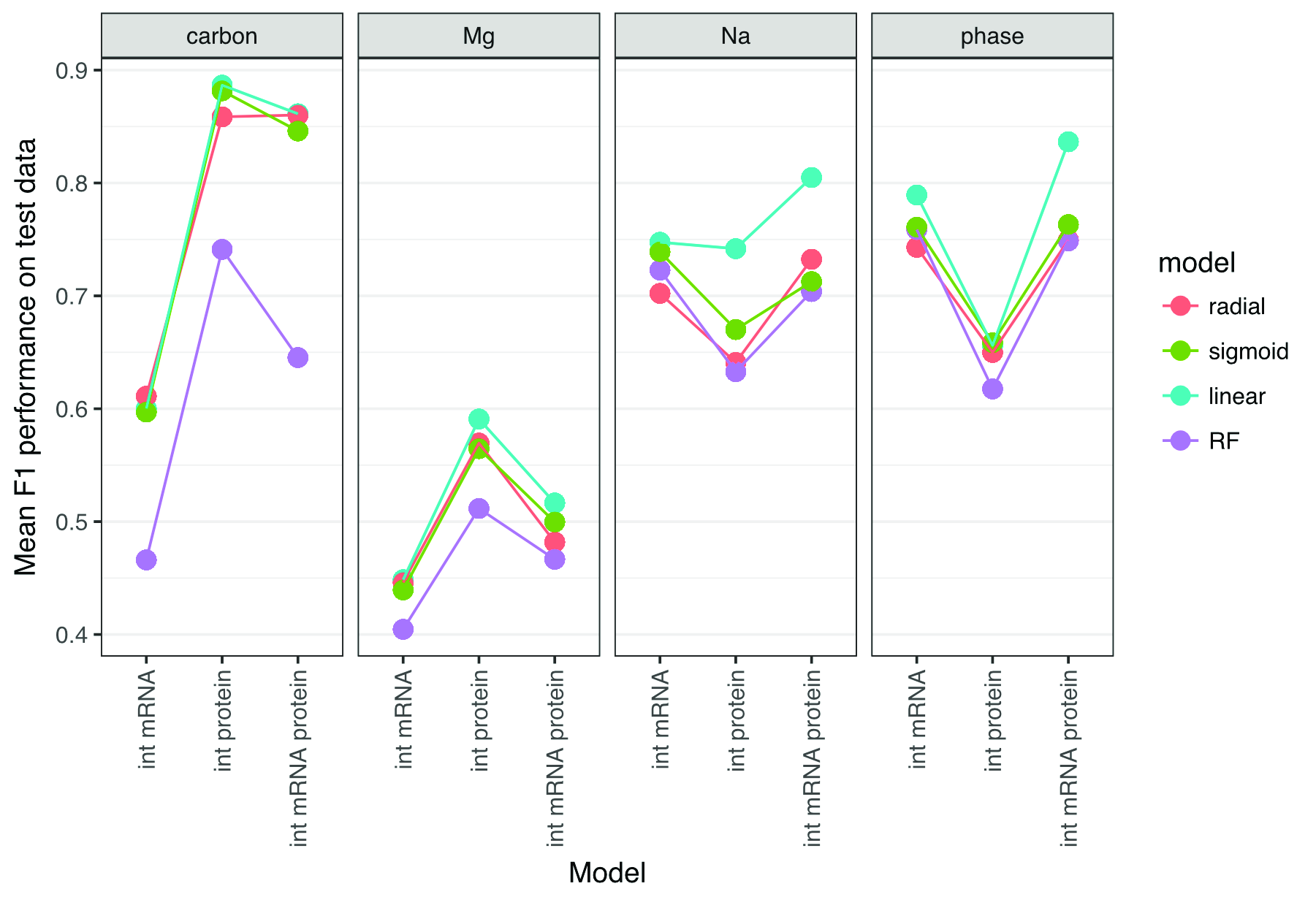

Supplement: S9 Fig — Protein abundances are more predictive for carbon source and Mg2+ levels, and mRNA abundances are more predictive for Na+ levels and growth phase. (TIFF) [file pone.0206634.s010.tiff]
